# Exploring cultural and linguistic influences on clinical communication skills: a qualitative study of International Medical Graduates

**DOI:** 10.1186/s12909-016-0680-7

**Published:** 2016-06-10

**Authors:** Anju Verma, Ann Griffin, Jane Dacre, Andrew Elder

**Affiliations:** Honorary Clinical Research Fellow, University College London Medical School, Royal Free Campus, Rowland Hill Street, Hampstead, NW3 2PF UK; UCL Medical School, University College London Medical School, 74 Huntley Street, London, WC1E 6AU UK; Royal College of Physicians, 11, St. Andrew’s Place, London, NW1 4LE UK; Edinburgh Medical School, 47 Little France Crescent, Edinburgh, EH16 4TJ UK; MRCP(UK) Federation of the UK Royal Colleges of Physicians, MRCP(UK) Central Office, 11, St. Andrew’s Place, London, NW1 4LE UK

**Keywords:** IMG, Cultural dimensions, MRCP(UK) PACES, Hofstede, Communication skills, Differential attainment

## Abstract

**Background:**

International Medical Graduates (IMGs) are known to perform less well in many postgraduate medical examinations when compared to their UK trained counterparts. This “differential attainment” is observed in both knowledge-based and clinical skills assessments. This study explored the influence of culture and language on IMGs clinical communication skills, in particular, their ability to seek, detect and acknowledge patients’ concerns in a high stakes postgraduate clinical skills examination. Hofstede’s cultural dimensions framework was used to look at the impact of culture on examination performance.

**Methods:**

This was a qualitative, interpretative study using thematic content analysis of video-recorded doctor-simulated patient consultations of candidates sitting the MRCP(UK) PACES examination, at a single examination centre in November 2012. The research utilised Hofstede’s cultural dimension theory, a framework for comparing cultural factors amongst different nations, to help understand the reasons for failure.

**Results:**

Five key themes accounted for the majority of communication failures in station 2, “history taking” and station 4, “communication skills and ethics” of the MRCP(UK) PACES examination. Two themes, the ability to detect clues and the ability to address concerns, related directly to the overall construct *managing patients’ concerns*. Three other themes were found to impact the whole consultation. These were building relationships, providing structure and explanation and planning.

**Conclusion:**

Hofstede’s cultural dimensions may help to contextualise some of these observations. In some cultures doctor and patient roles are relatively inflexible: the doctor may convey less information to the patient (higher power distance societies) and give less attention to building rapport (high uncertainty avoidance societies.) This may explain why cues and concerns presented by patients were overlooked in this setting. Understanding cultural differences through Hofstede’s cultural dimensions theory can inform the preparation of candidates for high stakes bedside clinical skills examinations and for professional practice.

## Background

International Medical Graduates (IMGs) are known to perform less well in many postgraduate medical examinations when compared to their UK trained counterparts [1–3]. This “differential attainment” is observed in both machine-marked knowledge based and bedside clinical skills assessments but has been of particular concern in the UK based Royal College of General Practitioners Clinical Skills Assessment (CSA) [4, 5].

The Membership of the Royal College of Physicians (UK) (MRCP(UK)) Practical Examination of Clinical Examination Skills (PACES) examination is the largest high stakes postgraduate clinical skills examination in the world with 5000 candidates examined each year. In this examination, which is conducted in the English language only, UK trained medical graduates (UKMGs) had a mean pass rate of 63.4 % between 2009 and 2013 while that of IMGs was 31.4 % [6].

PACES has a highly structured competency based assessment structure in which seven specific clinical competencies or “skills” are individually assessed [7]. IMGs score less well than UKMGs in all seven skills assessed but the difference in performance is greatest for skills that involve communication. One specific communication skill assessed in PACES is the ability of the candidate to “*manage patients’ concerns”*. This is described as *“seeks detects, acknowledges and attempts to address a patient’s or relative’s concerns. Listens. Confirms patient’s or relative’s knowledge and understanding. Empathic.”* This aspect of communication arguably has its origins within a Western approach to the doctor-patient relationship under the umbrella term “patient-centred care” and may be a less prominent feature of medical communication in other cultures.

Other research has identified communication differences between doctors from different cultural backgrounds that may impact on the patient-centred approach. A tendency to be paternalistic towards patients, family involvement in patient care [8–13] and concerns about encroaching on gender or social boundaries may make it difficult for some IMGs to offer emotional support to patients [14, 15]. Finally, different cultural perceptions of disease concepts such as depression in later-life and different cultural approaches to teaching and learning have also been identified [16]. McCullagh identified further challenges in communication for IMGs, such as understanding “patient speak” (colloquial language), developing awareness of politeness and respect, non-verbal communication, impact of culture and reflective practice [17].

A number of healthcare studies have utilised Hofstede’s cultural dimensions framework to look at the impact of culture on medical practice [18, 19]. Hofstede [20] defines culture as “the collective programming of the mind which distinguishes the members of one group from another”. The Hofstede framework categorises aspects of cultural behaviour across countries so that they can be measured and compared. The Hofstede framework has been applied in studies of cross-national differences in antibiotic use [21], blood donation [22], medical communication between general practitioners and patients [23] and the meaning of medical professionalism [24]. Morrow and colleagues [25] have used Hofstede’s framework to help understand adaptations that overseas trained doctors have to make when coming to work in the UK.

The framework originally consisted of four dimensions: power distance (PDI), uncertainty avoidance (UA), individualism/collectivism (IDV) and masculinity/femininity (MAS) [18, 19]. The definitions of these can be seen in Table 1.

**Table 1:** Four cultural dimensions, derived from Hofstede (2011)

Dimension	Definition	Countries
Power distance	Relates to the unequal distribution of power in a society and the degree to which less powerful members accept this. People in societies exhibiting a large degree of power distance accept a hierarchical order in which everybody has a place and which needs no further justification. In medicine doctors are seen as ‘God-like’ and the consultations are controlled by the doctor. In low power distance societies patients treat doctors as equals.	Higher power distance scores are seen in Eastern European, Latin, Asian and African countries.
		Lower scores are seen in Germanic and English-speaking Western countries
Uncertainty avoidance	Reflects the extent to which members of a society attempt to cope with anxiety by minimising uncertainty. Uncertainty avoiding cultures feel threatened by unknown or unstructured situations. They try to prevent such situations from arising by having strict laws, rules and behaviour codes. In medicine it has been shown that strong uncertainty avoiding societies place less emphasis on building rapport with patients.^23^	Uncertainty avoiding scores are higher in Latin countries, Japan, countries in East and Central Europe and those that are German speaking.
		Lower scores are seen in English speaking, Nordic and Chinese culture countries
Individualism vs. Collectivism	This the degree to which people in society are integrated into groups. A culture is deemed ‘Individualist’ if the members of the group are supposed to care for themselves and their immediate family only; the ties between individuals are loose. ‘Collectivist’ culture refers to societies in which people from birth onwards are integrated into strong, cohesive in-groups, which throughout a person’s lifetime continue to protect them in exchange for unquestioning loyalty.	Individualism is seen in the UK and other developed and Western countries.
		Collectivism prevails in less developed and Eastern countries, with Japan taking a middle position on this dimension
Masculinity vs. Femininity	This looks at differences between male and female values along a scale from “assertiveness and competition” to “caring and modest”. In masculine cultures the differences between gender roles are more dramatic such that men should be seen as assertive and ambitious, whilst women may not have these qualities. In feminine cultures greater emphasis is placed by both men and women on relationships and quality of life. Masculine cultures see open displays of competition between students who make themselves visible in class and over-rate their own performance	High masculinity scores are seen in the UK, Japan, German speaking countries and Mexico.
		Low scores are seen in Nordic countries and in the Netherlands and moderately low in some Latin and Asian countries like France, Spain and Thailand

This study is the first to use this framework in the exploration of candidate’s performance in a clinical examination in medicine. The aims of this qualitative paper, which forms one part of a larger project were (1) to identify the communication issues relating to the construct “*managing patients’ concerns”*, in IMGs failing the MRCP(UK) PACES examination in the UK, and (2) attempt to understand and explain this using Hofstede’s cultural dimensions framework.

## Methods

The methodological approach was qualitative and interpretive. It was a small-scale study exploring in detail communication failures of IMGs in an exam setting.

### Context

A single MRCP (UK) PACES examination site accustomed to video recording for quality assurance purposes was chosen for the study. The PACES examination consists of a carousel of eight doctor - patient encounters, all of which are directly observed by pairs of clinician assessors. This study focussed on the two stations in which communication skills are assessed in isolation from physical examination and where the skill of “*managing patients’ concerns”* is specifically assessed - station 2 (history taking) and at station 4 (communication skills and ethics). At these stations, trained actors (simulated patients (SP)) play the part of patients or their relatives and candidates are aware that one part of their task is to demonstrate the ability to detect, recognise and *manage patients’ concerns*. Two examiners, senior clinicians in the UK, directly observe and assess the candidate – SP interaction over a 14 min period.

### Data collection

Video and audio was chosen as the recording medium to capture spoken and non-verbal communicative aspects of the stations. Data were collected between October 2012 and November 2012.

All candidates listed to sit the PACES examination over a two week period in the examination centre chosen for the study were mailed a month before the examination with information about the project, and an invitation to take part. On the day of the examination, full written consent was sought from candidates who had indicated an initial interest. All those that had consented were video-recorded at station 2 and station 4 over a two-week period in 2012. As one aspect of the study intended to focus on candidates who failed the examination, and this could not be known before the examination, 36 IMG’s performances were recorded. 28 IMGs failed “managing patients concerns” of which 21 outright failed (scored zero). The qualitative analyses of the recordings were related to these 21 candidates who failed the examination overall in addition to outright failing the “managing patients’ concerns” section at stations 2 and 4.

### Analysis

The analysis focused on station 2 “history taking” and station 4 “communication skills and ethics” in which candidates’ ability to *manage patients’ concerns* was one of several skills explicitly assessed. A purposeful sample of 10 video recordings was made. It consisted of IMG doctors, for whom self-declared primary medical qualification (PMQ) details were available, who failed the examination outright in addition to failing the specific skill of *managing patients’ concerns* at these two stations. A deductive and inductive thematic content analysis approach [26] was chosen as it permitted concepts from Hofstede framework to be applied but also allowed the emergence of new themes. The recordings were transcribed verbatim. *QSR NVivo 10*^©^ software was used for data management.

Communication behaviour was analysed with reference to the MRCP(UK) definition of *managing patients’ concerns* and the following specific constructs:

- Seek concern – *doctor actively seeks out whether the patient has concerns.*
- Detect concern – *doctor picks up cue(s) that leads to uncovering a concern.*
- Acknowledge concern – *doctor expresses recognition of a concern.*
- Address concern – *doctor deals with the concern.*
- Confirm understanding – *doctor checks what patient has understood by using strategies such as asking the patient to restate in their own words.*
- Demonstrate empathy – *doctor communicates identification, understanding and/or appreciation of the patient’s situation.*

A single observer (AV) undertook all initial analysis. To ensure the reliability of the analysis, sections of coded data were reviewed by a medical educator with experience of teaching and assessing medical communication skills (AG). Themes were then developed and reviewed by the research team at a data validation workshop until consensus was reached. As coding progressed, new themes were created and existing codes themes refined. No new themes emerged after the first eight recordings.

Ethics approval was granted by the Institute of Education Research Ethics Committee and permission for the study was obtained from the then Medical Director (JD) and Chair of the Clinical Examining Board of MRCP(UK) (ATE). The research was conducted in accordance with British Educational Research Association guidelines [27].

## Results

The recorded consultations of 10 IMG candidates who failed the examination outright in addition to failing *managing patients’ concerns* were analysed in detail. Five of the IMG candidates had already attempted the examination at least once; all ten were aged 30 years or over and seven of the ten were male. Detailed demographic data are shown in Table 2.

**Table 2:** Demographics of 10 IMG candidates who failed at the observed attempt

Age	Gender	Primary medical qualification	Self-declared Ethnicity	Attempt at exam
30	M	Malaysia	Chinese/Chinese British	2
44	M	Indonesia	Chinese/Chinese British	1
36	F	India	Indian	4
36	M	India	Unknown	1
33	M	Pakistan	Pakistani	5
34	F	Myanmar	Asian – Other	1
41	M	Egypt	Arabic	1
42	M	Pakistan	Pakistani	5
42	M	Pakistan	Pakistani	5
30	F	Pakistan	Pakistani	1

Table 3 shows the characteristics, in relation to Hofstede’s original four dimensions, of individuals from the countries from which the candidates in this study had gained their primary medical qualification.

**Table 3:** Countries of candidates’ primary medical qualification: Scores on Hofstede’s cultural dimensions relative to Great Britain (Source: http://www.geerthofstede.eu/dimension-data-matrix)

	PDI	*relative*	IDV	*relative*	MAS	*relative*	UAI	*relative*
Great Britain	35	*0*	89	*0*	66	*0*	35	*0*
Arab speaking countries (Egypt, Iraq, UAE)	80	*45*	38	*-51*	53	*-13*	68	*33*
India	77	*42*	48	*-41*	56	*-10*	40	*5*
Indonesia	78	*43*	14	*-75*	46	*-20*	48	*13*
Malaysia	104	*69*	26	*-63*	50	*-16*	36	*1*
Myanmar	No data	*No data*	No data	*No data*	No data	*No data*	No data	*No data*
Pakistan	55	*20*	14	*-75*	50	*-16*	70	*35*

*PDI* Power distance index, *IDV* Individualism/collectivism, *MAS* Masculinity/femininity, *UAI* Uncertainty avoidance

Great Britain has been used to provide a comparator and the relative and absolute scores on a continuum between 0 and 100 of the other countries are shown (where these data were available) [28]. The higher the score, the higher the power distance, uncertainty avoidance, individualism and masculinity.

A list of five coding themes emerged from transcription analysis. Two themes directly related to the constructs of *managing patients’ concerns* (detecting clues and concerns and addresses concerns). Three themes: building relationships, providing structure to the consultation and explanation and planning impacted on the whole consultation. These are elaborated below.

### Theme one: Detects cues or concerns

A particular feature of the analysis was that IMGs tended to miss or overlook cues and concerns, a case involving a female patient in her 60s who presents with weight loss highlights this and the candidate appears not to have heard the patient explaining how tired she feels.

*Pt “And during the day it’s not so much but mostly it’s the tiredness really. It’s**** really ****just zapping me”.*

*IMG 15 “So you were fine before the five or six weeks and now you have fever, night sweats, and weight loss. Do you feel tired?”*

Another IMG seems to pick up on some cues but doesn’t follow these up with relevant questions. For example, the candidate starts to ask about her work but follows this with a new line of questioning:

*Pt “I work all over the place actually. I do a lot of travelling around.”*

*IMG 11 “Hmm…Another thing, which I have noticed, is you are smoker. You are a smoker.”*

### Theme two: Addresses concerns

This theme focused on whether or not IMG candidates attempted to address concerns after the patient had raised them. In most cases they were not addressed directly, and sometimes, as the consultation below highlights not at all.

*Pt “And it just seems, I just aged like an old woman. I’m just exhausted. And what’s in my head is cancer. It’s the only thing I can think about. Do you think that’s what’s wrong?”*

*IMG 15 “Um, do you smoke cigarettes before?”*

*Pt “Do I smoke? I don’t smoke.”*

Here the candidate does not acknowledge the patient’s concern of cancer. The consultation continues:

*IMG 15 “So any family history of the cancer or anything like that before? ”*

*Pt “No, not that I’m aware of. What do, do you think this is what it is?”*

*IMG 15 “No, actually, from the history I have taken and the received this blood tests, I’m thinking of some blood related problems.”*

When the patient tries to clarify what the doctor means she encounters medical jargon:

*Pt “What, what do you think it is?”*

*IMG 15 “Um, probably related to the blood. Your haemoglobin is quite low. And, your ESR, that’s a marker of some inflammations in baseline, some disease activity, some certain disease activities, is quite high, as well. And your symptoms like weight loss,”*

*Pt “Yes”*

*IMG 15 “Fever, and night sweats. This quite high… some problems with your blood.”*

### Theme three: Building relationships

This theme concerns the ability to develop rapport with the patient by using and detecting non-verbal behaviour to guide the consultation, using empathy to communicate understanding and appreciation of the patient’s feelings or situation and sharing thinking and rationale with the patient. It also involves the doctor taking responsibility for his or her actions and being the patient’s advocate.

This theme is highlighted with reference to the following PACES scenario: A pregnant patient attends for an antenatal ultrasound scan. All is well with the baby, but cysts have been discovered in her own kidneys, indicating a diagnosis of ‘adult polycystic kidney disease’. This is an inherited condition which she may pass on to her unborn child. 10 % of patients require dialysis in the future.

The candidate starts off by asking an introductory question that is difficult for the patient to answer:

*IMG 27 “So, I saw the letter, so umm, can you tell me how are you and what’s going on in your life?*

*Pt “Umm… …What’s going on generally?”*

*IMG 27 “Generally in your? What bothers you? We are here, why we are here. Do you?”*

The SP mentions the concerns about the scan. Although the candidate tells the SP there is nothing for her to worry about regarding the baby, the candidate begins to repeat themselves saying ‘don’t worry about the baby’, ‘cysts were found in your kidneys’, ‘your kidney function is normal’ without further explanation. The candidate attempts to seek out concerns *after* saying to the patient that she must be worried and the SP’s frustration is clearly seen when she responds with a strong use of body language:

*IMG 27 “And for that purpose I know you must be worried. What are your worries at the moment?”*

*Pt “Well obviously I’m worried about my baby and the pregnancy, but you’re telling me that’s all fine. I’ve nothing to worry about.”*

*IMG 27 “Nothing to worry for the baby, no. Okay? Nothing to worry for-”*

*Pt “I’ve got that! {The patient pushes both hands out in front of her with the palms facing the doctor} There’s nothing, right, so. But there’s, you’ve found something on my kidneys.”*

The candidate then tells the patient “don’t worry” eight times and dominates the consultation.

Overall, the IMG candidates in this study did not involve the patient in discussions about their care and used the phrase “I told you” a number of times. IMGs tended to interrupt the patient a number of times, (indicated by -)and there were many instances of overlapping speech as indicated by [].

*Pt “Okay. So what are the other symptoms then? What should I be [looking out for?]”*

*IMG 27 “[You want to now] right now is it though? Nice time that you want to know? I know it must be”*

*Pt “[Well it’s-]”*

*IMG 27 “[difficult for] you to absorb also. We can have another meeting to discuss about these symptoms also.”*

*Pt “Well, I would like to know the symptoms [today]”*

*IMG 27 “[You want] to know the symptoms?”*

*Pt “Yes. I need to know abou-”*

*IMG 27 “Sometimes its affects the heart, sometimes there is you know enlargement of the liver also.”*

### Theme four: Providing structure to the consultation

This theme was based on the idea that effective consultation requires a logical sequence to asking questions or providing explanations, and using signposting and summarising to help move the interview along. The IMGs generally adopted a rigid style, asked a lot of questions, and often did much of the talking. IMG candidates often asked several questions at once such that it was not always clear which question the patient had answered.

*IMG 15 “Okay. Have you ever had any other problems apart from asthma, high blood pressure? Any blood test check before? Anaemia or anything like that?”*

*Pt “No, I mean my GP just checked my bloods and said they were fine.”*

And in another scenario

*Pt “I just smoke a little bit.”*

*IMG 11 “And since when, and how much? Have you ever thought about quitting it?”*

*Pt “I’ve only just started smoking really. Not long ago.”*

### Theme five: Explanation and planning

This theme is based on the part of the consultation in which the clinician aims to provide the correct type and amount of information to the patient [29]. Two approaches were taken, one where large amount of information containing medical jargon was given, or explanations would be insufficient and vague. There was little involvement of the patient in the discussion, specifically exploring what the patient already knew, and the candidate tended to tell the SP what to do. These points are highlighted in a scenario involving a forty-five year old woman who was admitted with a severe attack of asthma. She had not been using her inhalers and was going through a stressful period in her life. She also smoked.

*IMG 10 “The other factor that can worsen your asthma is smoking cigarettes. So you, uhh, need to cut down on all these trigger factors. Obviously, I think you are more concerned it may happen to you again, is one of your concerns, isn’t it?”*

*Pt “Yeah, of course.”*

*IMG 10 “So we need to make sure that, you know, couple of things like ensuring compliance with the medication, you need to go strictly on your asthma inhalers, and you need to take these regularly as well, and so these are the views of-”*

*Pt “But if I don’t feel that it’s helping, why would I take it?”*

*IMG 10 “Umm, one thing is that, you know, all the medications that we have given you should be taken in regular dosages. And there’s a protocol for these. I mean, obviously, we can escalate the thing as well for you, the dosages wise. And you, we always start the people on low dosages of the medication.”*

Medical terminology was used and there was inadequate explanation for the patient.

### All themes illustrated: Managing Patients’ Concerns during “breaking bad news” of a cancer diagnosis

Although the five themes have been presented as distinct entities they are often overlapping and this is illustrated with examples from a breaking bad news scenario. Candidates tended to lack explicit signposting, often referred to as the ‘warning shot’ and there was also little evidence of balancing hope with reality.

In one scenario, a female patient with a past history of breast cancer was admitted to hospital six weeks ago with an asthma attack. Whilst in hospital she underwent a chest x-ray, the results of which are suspicious of cancer having returned and spread. The patient is unaware of the findings. She attends for a follow up appointment in the out-patient clinic. One IMG starts to explain the x-ray findings and delivers the news swiftly:

*IMG 1 “…I’m here to discuss with you to explain to you the x-ray.”*

*Pt “Uhh hum”*

*IMG 1 “And are you ready to, to listen to the, to my report? Anyone with you? You came alo-, are you coming alone?”*

*Pt “Yeah umm, yes.”*

*IMG 1 “Ahh okay. Sorry. So from the x-rays that was taken six weeks ago and err, we noted that we found that the presence of two white spots in your right lungs.”*

This IMG tended to give the patient little time to process the possibility of cancer having returned:

*IMG 1 “Okay. Now I would like to explain to you…regarding the white spots. On this current x-ray we are susp- suspecting that it could be something that is not good, something noxious. It put in another way it could be a form of…cancer.”*

*Pt “What I’ve got the, you think its cancer?”*

*IMG 1 “We couldn’t rule out at this moment.”*

*Pt “So, you think this is the, it’s got nothing to do with the asthma? It’s the cancer?”*

In general, IMGs tended to use more medical jargon for example, again from the same scenario:

*IMG 1 “As I said mentioned earlier on we have ordered you a C- imaging a further test in the form of imaging.”*

*Pt “Right [So what’s?]”*

*IMG 1 “[Its a, we] call it CT scan”.*

*Pt “Right”*

*IMG 1 “A CT scan is a form of…it’s…it works on the principles of an x-ray but it’s magnified. It’s have magnified intensities. Many of these intensities are x-ray beams.”*

Whilst breaking bad news, some IMG candidates avoided using the word cancer. Instead they referred to “growth”, “lesion” or “tumour”. Furthermore, IMG candidates also tended to look down or away from the patient during sensitive parts of the consultation, such as when the patient spoke about their worries or asked directly if the doctor thought the problem may be cancer.

*IMG 8 “…and when we done the MRI scan unfortunately there wasn’t what we expected I’m afraid. They showed that some of the growth in the brain.”*

*Pt “So, I’m sorry/a growth [in the brain?]”*

*IMG 8 “It’s [like a, yes]”*

*Pt “What does that mean?”*

*IMG 8 “I mean we call as a meta- mets. I don’t know whether you are understand the term? Do you know what that, have you been?”*

*Pt {Patient shakes her head to signal no}*

*IMG 8 “Because, sometimes what happens, we call it a tumour, an abnormal growth in the brain.”*

One particular IMG doctor continues in this manner, explaining the spread of cancer as “come from other place” until she mentions involving “cancer doctors” ten minutes into a fourteen-minute consultation:

*IMG 8 “…the doctors going to help you, the doctors that are dealing with the tumour, what are, who are the cancer doctors. And they will come and talk to you what type of the management we are going to do, what kind of-”*

*Pt “Well, I, I. You’ve said that word now; I was kind of hoping to avoid that word. Is that what you think this is?*

*IMG 8 “What, do you mean by…cancer?”*

## Discussion

### Summary of findings

In this study distinct reasons were found in the communication skills demonstrated by IMGs sitting the MRCP(UK) PACES examination which explained reasons for their failure in the skill of *managing patients’ concerns* and, more broadly, their ability to communicate in a general sense throughout the consultation. The themes identified in this study relate in particular to Hofstede’s cultural dimensions of power distance, uncertainty avoidance and masculinity/femininity (Table 1) and are discussed in detail below.

### Power distance index

Characteristics of high power distance societies came across in the consultation styles of the IMGs in the themes ‘explanation and planning’ and ‘providing structure to the consultation’ which impacted on ‘building relationships’. IMG doctors appeared to control the consultation either by interrupting the patient, or taking responsibility away from the patient. The IMG doctors’ thought processes were not made explicit and the communication tended to be one-way. These findings are in keeping with Meeuwesen et al.’s cross-national study of communication between GPs and patients [23]. They found that the larger a nation’s power distance the more fixed the roles of the doctor and patient, and the less room there was for unexpected information exchange: an attitude described as “doctor knows best” prevailed.

Hofstede reports that in high power distance countries there is more reluctance to disagree with or question those in charge [18]. IMGs who have trained in high power distance countries are more likely to see themselves in a position of responsibility, and more dissociated from the patient, as this is the cultural norm for them. Morrow et al. [25] found that the difference in power distance in the doctor - patient relationship was the largest difference reported by IMGs in their transition to working in the UK. IMGs from high power distance countries tended to position themselves as the experts. Although this may not provoke difficulty if both doctor and patient are from high power distance countries, this may not be the case when there is power-distance mismatch. Patients from lower power distance countries, as in this study, are more inquisitive and expect to be involved in their treatment and this may prove challenging to the doctor.

IMGs in this study had difficulties detecting patients’ cues and addressing their concerns. This may suggest that these features are not a usual or expected aspect of medical consultations in those from high power societies. The hierarchical nature of high power distance societies may also encourage a more rigid style of consulting with little flexibility in the order of questioning as was observed in the current study.

### Uncertainty avoidance

High uncertainty avoiding countries tend to pay less attention to building rapport. This can be seen in the theme ‘building relationships’ and the difficulties observed with ‘detecting cues’ and ‘addressing concerns’. In a previous study, candidates from countries with a higher tolerance for uncertainty, had more eye contact and paid more attention to rapport building [23]. In the current study IMGs, from countries with lower tolerance of uncertainty, tended not to answer questions posed directly to them, which often crystallised the patient’s concerns, and this may reflect difficulties in expressing diagnostic uncertainty. This has also been observed in a study on antibiotic prescribing, in which doctors from higher uncertainty avoiding countries issued more antibiotic prescriptions for self-limiting viral illnesses than those from lower uncertainty avoiding countries. This was suggested to mean that a “wait-and-see” or expectant approach would be interpreted by the patient as meaning that the doctor did not know the right answer [21]. In the PACES scenarios studied there was not necessarily, by design, any immediately available correct option or answer to the issues raised, and some IMGs appeared uncomfortable when difficult questions were posed to them, sometimes choosing not to answer the question at all. This may explain why IMGs generally tended to avoid the word “cancer”. The absence of a definitive diagnosis may create more uncertainty than some doctors are comfortable with, and avoiding any words which suggest cancer, or the word cancer itself, is a plausible method of minimising uncertainty. Asking patients’ opinions on management options, a central component of a “patient - centred” consultation, could be construed in a similar manner and may explain why IMGs tended not to use this approach.

IMGs generally used a didactic style of conversation with patients and rapport also appeared to be hampered when an attempt to pick up cues from the patient, to which a patient responded, were not then fully explored. In consultations in which this was observed IMG doctors appeared to become fatigued by a patient-centred style of consulting and, perhaps simply because of pressure of time, reverted back to the doctor-centred style which may be more natural for them. If this is the case, those from high uncertainty avoiding societies may have more difficulty in building relationships in brief, time-pressured consultations, as the flexibility of a more patient-centred approach is outside their cultural norm. The more rigid consulting style often adopted by IMGS in this study appeared to make it more difficult to provide detailed explanation or approach shared planning with patients.

### Masculinity/Femininity

This dimension is associated with smaller differences in Hofstede scores across the range of countries of PMQ of the IMGs in this study (Table 3). IMG countries typically cluster around the middle of the range and the UK tends towards a more “masculine” society. Candidates may thus be expected to demonstrate fewer appreciable cultural differences on this dimension.

The Hofstede framework suggests that doctors from more masculine societies may be expected to use more disease focused biomedical language, and place more emphasis on solving problems or curing patients. However, the IMG doctors in this study also used a significant amount of biomedical language. The impact of a candidate’s own sex upon these behaviours is not possible to ascertain but may be relevant. Meeuwesun et al. [23] also observed that disease oriented language was seen to be used in more *feminine* cultures and that IMGs also tended to omit the patient’s understanding, ideas, concerns and expectations, as was found throughout this study.

### Strengths and limitations

This is the first study to explore Hofstede’s framework in a high stakes postgraduate clinical examination setting. There are a number of strengths and limitations to the current study. Firstly, the sample size of 10 video recordings is relatively small. The sample was also from a single examination site; consequently, the data may not be representative of a wider population.

However, a sample recruited to a qualitative study is not usually intended to be representative of a larger population or to produce data that can be widely generalised. More important is the notion of ‘transferability’ of findings [30] through the use of a theoretical framework.

Secondly, it is important to note that Hofstede’s framework applies at a national or group level, rather than at the level of the individual and country-level preferences cannot be assigned to individual behaviour. Furthermore, Hofstede has not actually researched dimensions of *medicine* as a sub-culture within any one country. Despite the typical use of the Hofstede framework at an organisational level, this study has demonstrated its utility as a tool to interpret cultural factors at an individual level.

Purposive sampling was used to maximise diversity in approaches to *managing patients’ concerns* in IMGs from a range of cultural backgrounds. Additionally, saturation was reached as no new themes emerged after analysis of eight recordings for the group.

## Conclusions

In this study, IMGs who failed MRCP (UK) PACES, an examination created and assessed in the UK, and designed to ensure competence of practice in a UK setting, demonstrated consultation skills that were culturally different to the norms expected. The findings parallel differences that have been identified amongst IMGs working in the UK [12, 25]. Future research could investigate whether similar findings are echoed across all stations of the PACES examination and analyse a broader group of candidates from multiple examination centres.

Hofstede’s framework may help us understand nations’ cultural norms and values in relation to examination performance and professional practice. In particular, power distance and uncertainty avoidance dimensions may predict areas where IMGs are more likely to have difficulty, and provide a rationale for why this may be the case. An understanding of this may help IMGs to understand what is meant by patient - centred care in UK medical practice, and to reflect on their earlier training environments and the effects of the cultural norms embedded within those environments on their current practice. An understanding of these differences may help us to narrow the attainment gap between UK Medical Graduates and IMGs in clinical and professional medical examinations. Our findings are likely to be of value to all doctors sitting the MRCP (UK) PACES examination, and be of interest to other Royal Colleges and their candidates who sit their face-to-face examinations in the UK.

## Abbreviations

CAS, Clinical Skills Assessment; IDV, individualism/collectivism; IMG, International Medical Graduates; MAS, masculinity/femininity; MRCP, Membership of the Royal College of Physicians; PACES, Practical Examination of Clinical Examination Skills; PDI, power distance; PMQ, Primary medical qualification; SP, Simulated patient; UA, uncertainty avoidance; UKMG, UK trained medical graduates
